# Change in alcohol consumption and all-cause mortality among postmenopausal women diagnosed with breast cancer

**DOI:** 10.1007/s10549-026-08113-1

**Published:** 2026-10-23

**Authors:** Samantha Rees, Clara Bodelon, Saptarshi Chakraborty, Amy E. Millen, Ying Wang, Kierstin Faw, Lauren R. Teras, Marissa M. Shams-White, Alpa V. Patel, Jo L. Freudenheim

**Affiliations:** 1 Department of Epidemiology and Environmental Health, School of Public Health and Health Professions, University at Buffalo, The State University of New York, 270 Farber Hall, 360 Hayes Road, Buffalo, NY 14214 USA; 2 Department of Population Health Sciences, British Columbia Cancer Research Institute, BC Vancouver, Canada; 3 Department of Population Science, American Cancer Society, Atlanta, GA USA; 4 Department of Actuarial Mathematics and Statistics, School of Mathematical and Computer Science, Heriot Watt University, Edinburgh, UK

**Keywords:** Breast cancer survivors, Alcohol consumption, Behavior change, All-cause mortality

## Abstract

**Purpose:**

Evidence is inconsistent regarding an association between alcohol consumption and survival following a breast cancer diagnosis; therefore, we examined how change in alcohol consumption affects all-cause mortality (ACM) in a population of breast cancer survivors.

**Methods:**

We prospectively assessed pre-diagnosis, pre- to post-diagnosis, and post-diagnosis change in alcohol consumption among 1,558 postmenopausal women (mean age-at-diagnosis: 71 years) diagnosed with non-metastatic invasive breast cancer from the American Cancer Society Cancer Prevention Study-II Nutrition Cohort (baseline: 1992–1993). Five categories of alcohol intake were determined at each assessment; change in alcohol consumption was categorized as stable, quit, reduced, and increased. Change occurred at a median of two years before (pre-diagnosis) and two years after (post-diagnosis) diagnosis. Cox proportional hazards regression was used to estimate adjusted hazard ratios (HR) and 95% confidence intervals (CI) for the associations between change groups and ACM.

**Results:**

Over a median follow-up of 12.6 years, 750 women died (130 breast cancer deaths). Compared to stable consumption, the association between cessation and ACM varied by change interval: pre-diagnosis (HR:0.91, CI:0.69–1.21), pre- to post-diagnosis: (HR:1.39, CI:1.12–1.73), post-diagnosis: (HR:0.98, CI:0.70–1.35). Alcohol reduction was not associated with ACM. Among women consuming ≥ 1 drink/day pre-diagnosis, increasing consumption elevated ACM risk (HR:1.83, 95%CI:1.02–3.31).

**Conclusion:**

Alcohol reduction or cessation was not associated with a decrease in ACM among older breast cancer survivors. Among those drinking ≥ 1 drink/day, an increase in consumption pre-diagnosis was associated with higher mortality. Findings may be limited by uncontrolled confounding, a low prevalence of heavy drinking, or an insufficient duration of sustained change.

**Supplementary Information:**

The online version contains supplementary material available at https://doi.org/10.1007/s10549-026-08113-1.

## Introduction

As the prevalence of breast cancer survivors increases [1], strategies aimed at improving quality of life and survival become increasingly important. Alcoholic beverage consumption is an established risk factor for breast cancer development [2–5] but studied far less in relation to survival [6]. Findings from studies exploring associations between alcohol consumption, at a single point in time, and survival are inconsistent. In several studies, there was no association between alcohol intake either before or after diagnosis and mortality [7–12]. In others, alcohol consumption around the time of diagnosis was associated with a lower risk of all-cause mortality (ACM) [12–14]. In one study, consuming at least six grams/day after a breast cancer diagnosis increased the risk of breast cancer-specific mortality [15]. Although most research on this question compares moderate drinkers to non-drinkers, this approach may produce biased effect estimates if former drinkers are included in the non-drinker category. Alcohol guidelines for breast cancer survivors currently mirror those for the general female population, except to limit drinking to reduce the risk of recurrence [16].

There are few studies examining the association of change in alcohol consumption over time with survival among women with breast cancer. Newcomb et al. examined change in alcohol consumption both before and after diagnosis among breast cancer survivors [8]. While they found no association with breast cancer-specific mortality, increased consumption was associated with lower ACM (HR:0.76, 95%CI: 0.60–0.97) [8]. However, their analysis used non-drinkers as the referent; to understand the effects of change in consumption, continued stable consumption is a more appropriate referent group. Furthermore, reduction and cessation are distinct behaviors with varying underlying motivations that are often not explored separately, limiting our ability to make reduction-specific recommendations.

The American Cancer Society Cancer Prevention Study-II (CPS-II) Nutrition Cohort has repeated measures of alcohol consumption collected over a 21-year period. We report here on change in alcohol consumption in association with ACM among postmenopausal women diagnosed with invasive breast cancer, examining change during three intervals—pre-diagnosis, pre- to post-diagnosis, and post-diagnosis.

## Methods

### Study design and population

The Cancer Prevention Study-II (CPS-II) Nutrition Cohort began in 1992–1993 as a prospective cohort study of cancer incidence and mortality. Extensive participant data were collected at baseline and in follow-up questionnaires (years: 1992, 1997, 1999, 2003, 2007, 2013). Informed consent was obtained at enrollment and applied upon completion and submission of follow-up questionnaires. IRB approval was obtained for all aspects of the CPS-II Nutrition Cohort from the Emory University Institutional Review Board.

This analysis included postmenopausal women diagnosed with invasive breast cancer who consumed alcohol prior to diagnosis with a sufficient number of alcohol consumption measures. Because majority of women were postmenopausal in the CPS-II Nutrition Cohort at baseline; we did not have power to explore these associations for pre- or peri-menopausal women. We excluded women who were not current alcohol consumers at baseline, without a verified invasive breast cancer diagnosis, who were not postmenopausal at the time of breast cancer diagnosis, or who were diagnosed with distant stage breast cancer. To reduce the likelihood of reverse causation, we excluded those who died within the first year of follow-up, which was either one year after diagnosis (pre-diagnosis interval) or one year after alcohol change occurred (pre- to post- and post-diagnosis intervals). The final analytic sample for each change interval excluded those missing exposure or covariate data (i.e., stage, education, age-at-diagnosis, pack-years smoked, comorbidity data, body mass index, and estrogen receptor status) at the corresponding time (Supplemental Materials, Fig. 1). For example, women without a baseline and at least one pre-diagnosis measure of alcohol intake were excluded from the pre-diagnosis change sample but may have been eligible to contribute to the other time intervals. Our final sample size included *N* = 1,330 (608 deaths) women in the pre-diagnosis change interval, *N* = 1,558 (750 deaths) for the change pre- to post-diagnosis interval, and *N* = 975 (467 deaths) for the post-diagnosis change interval, not mutually exclusive.

### Alcohol assessment

Alcoholic beverage consumption was self-reported up to six times in the CPS-II Nutrition Cohort, in the 1992 (baseline), 1997, 1999, 2003, 2007, and 2013 questionnaires. The reliability and validity of these alcohol data were found to be reasonably valid [17]. Frequency and quantity of drinks per drinking occasion over the prior year were assessed in each questionnaire; specific questions varied somewhat across years (*Supplemental Materials*,* Table 1*). We harmonized alcohol consumption data across questionnaires using a multistep approach: (1) one alcoholic drink was considered equal to 14 g of ethanol, (2) drinking frequency responses were converted into average daily proportions using category midpoints divided by 7 days per week, and (3) at each timepoint, alcohol consumption (g of ethanol/day) was further categorized as 0, >0-<0.5, 0.5-<1, 1-<2, and ≥ 2 drinks/day. In addition, the differences in very low and very high levels of alcohol consumption across questionnaires were minimized by excluding baseline 1992 non-drinkers and unifying upper limits into the ≥ 2 drinks/day category. Change in alcohol consumption was determined by comparing a participant’s first alcohol consumption measure to their second; if they reported consumption in a different category, they were categorized as having changed their intake (i.e., quit, reduced, increased). Stable consumption was defined as remaining in the same intake category at both times.

**Fig. 1:**
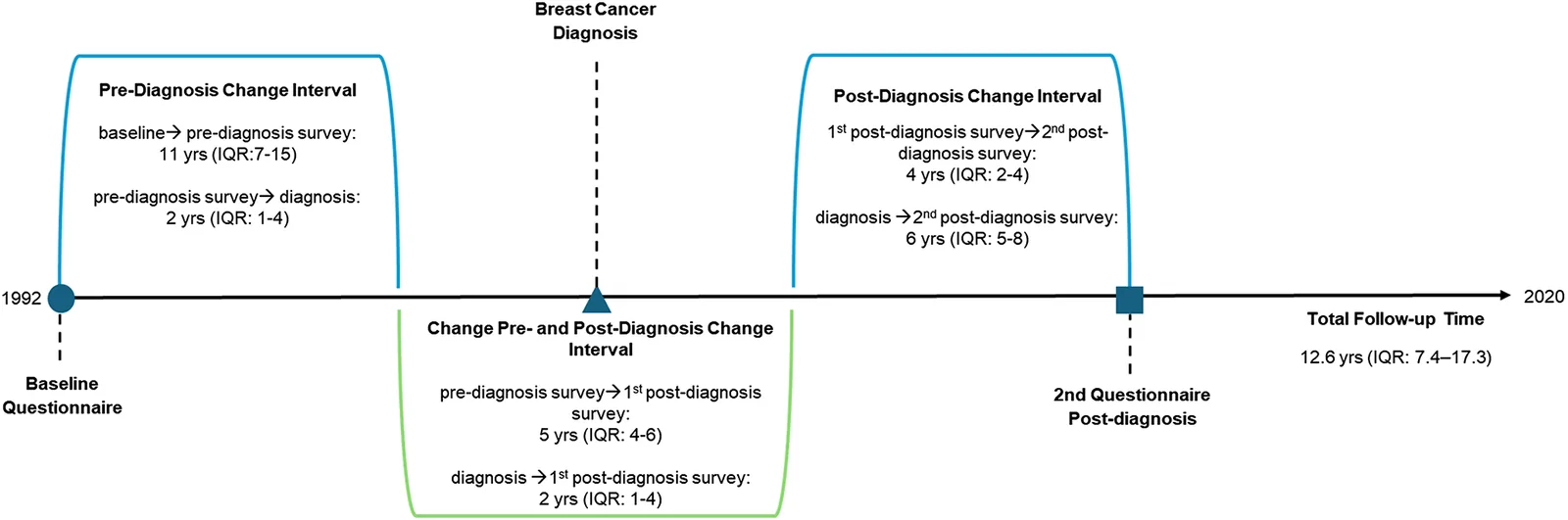
The three time intervals examined in relation to study timeline and breast cancer diagnosis; pre-diagnosis (*N* = 1,330), pre- to post-diagnosis (*N* = 1,558), and post-diagnosis change (*N* = 975)

Change in alcohol consumption was assessed at three different time intervals: before diagnosis (pre-diagnosis), before and after diagnosis (pre- to post-diagnosis), and after diagnosis (post-diagnosis) (Fig. 1). The years used for the measurement of change depended on when a participant was diagnosed with breast cancer. For example, the pre-diagnosis change interval baseline alcohol (1992) was compared to a participant’s last completed pre-diagnosis questionnaire, which had to be at least one year prior to diagnosis (median time: 2 years, interquartile range (IQR): 1–4 years). The last completed pre-diagnosis questionnaire was compared to the first post-diagnosis alcohol measure for the change pre- to post-diagnosis interval. Lastly, for post-diagnosis change, the first post-diagnosis measure was compared to the second post-diagnosis measure. The start of person-time depended on the change interval. For the pre-diagnosis interval, it began one year after breast cancer diagnosis; for the pre-to-post-diagnosis interval, it began one year after the first post-diagnosis measurement; and for the post-diagnosis interval, it began one year after the second post-diagnosis measurement.

### Outcome

The primary study outcome was death from any cause (ACM). Breast cancer-specific mortality was explored as a secondary endpoint. Vital status and cause of death were ascertained from the National Death Index (NDI) through December 31, 2020 [18]. All self-reported breast cancers and corresponding tumor data were verified (i.e., medical records, registry linkage, or NDI linkage). In total, fewer than 200 women died of breast cancer; breast cancer-specific mortality analyses are presented in the *Supplemental Materials* and should be interpreted cautiously.

### Statistical analysis

Cox proportional hazards regression was used to estimate hazard ratios (HRs) and 95% confidence intervals (CIs) for associations of change in alcohol consumption at the three change intervals with risk of ACM. To examine competing risks, Fine and Gray models were used for the breast cancer-specific analyses [19]. Women whose alcohol consumption did not change during the corresponding time interval (stable consumers) served as the referent group for each analysis. The proportional hazards assumptions were tested using Schoenfeld plots and residual-based tests; violations were handled using covariate-time interaction terms. All models were adjusted for prior alcohol intake (first alcohol measure for corresponding change interval, g/day), time between surveys, age at diagnosis (year), stage (localized or regional), ER status (ER+ or ER-), smoking (pack years*smoking status), comorbidities incident or at baseline (diabetes and/or CVD). Product terms were used to test multiplicative interaction between alcohol cessation and health status. Follow-up ended at death, loss to follow-up, or the study conclusion in 2020. We did not account for multiple comparisons, instead focusing on the magnitude of effect and corresponding uncertainty (95%CI). All analyses were performed in SAS version 9.4 (SAS Institute, Cary, NC).

Several sensitivity analyses were conducted. Further adjustment for BMI, education, or marital status did not meaningfully change the effect estimates and were therefore not included as confounders. To examine whether associations were impacted by uncontrolled confounding by health characteristics, subgroup analyses were performed: among never smokers, those without major comorbidities during the corresponding change interval, those whose perceived health status was good or excellent, and those with localized stage breast cancers. In addition, analyses restricted to participants drinking at least one drink/day were conducted to evaluate whether the magnitude of change impacted the observed associations. To assess the impact of the duration of change, we evaluated sustained post-diagnosis change, defined as participants who altered their drinking behavior after diagnosis and remained in that category of change at the second post-diagnosis measurement.

## Results

**Fig. 2:**
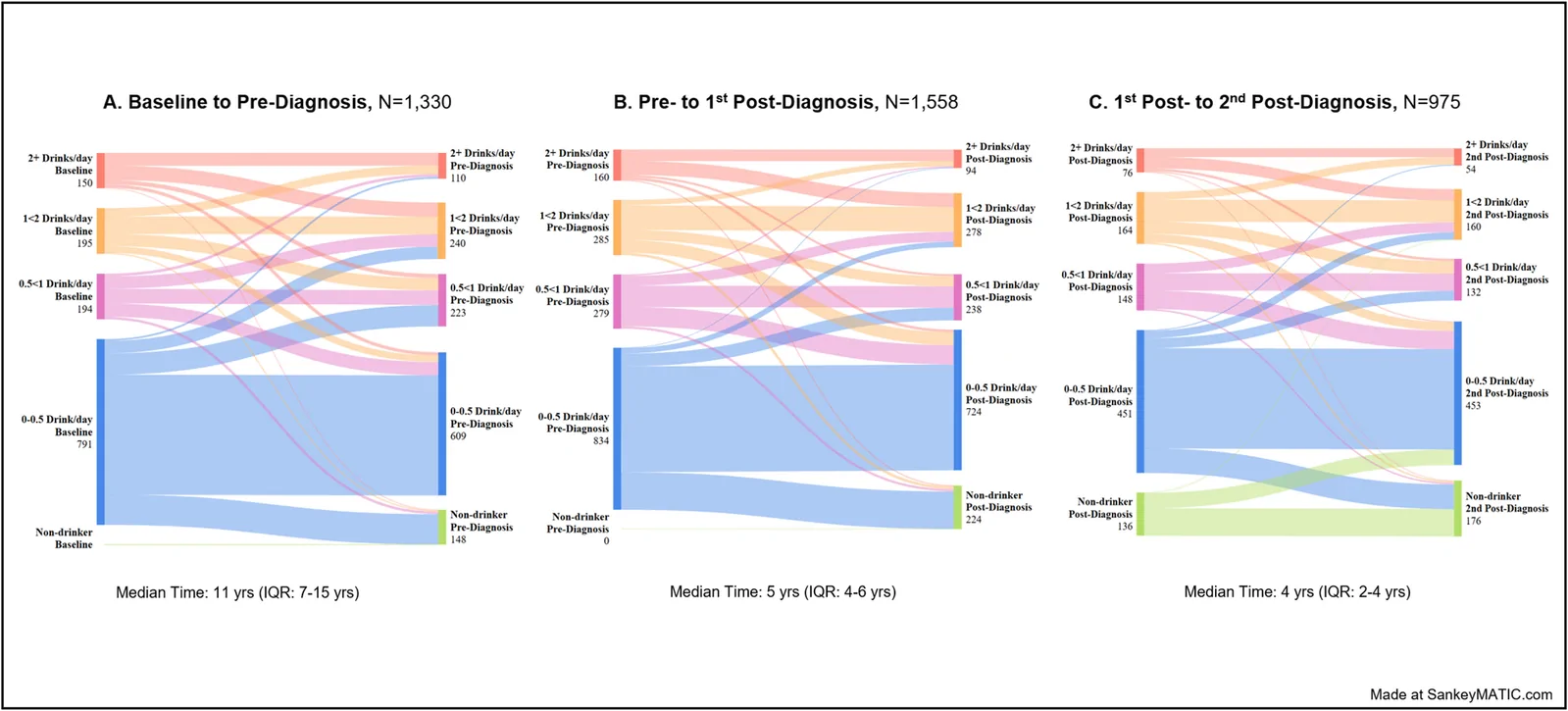
Change in alcohol consumption time intervals from (**A**) baseline to last pre-diagnosis, (**B**) pre-diagnosis to first post-diagnosis, and (**C**) first post-diagnosis to second post-diagnosis among postmenopausal breast cancer survivors

The three different intervals for change in relation to study timeline and breast cancer diagnosis are shown in Fig. 1. Total sample size, breast cancer case count, and person-time varied by change interval: pre-diagnosis (*N* = 1,330, 608 cases, and 17,059 person-years), pre- to post-diagnosis (*N* = 1,558, 750 cases, and 19,173 person-years), and post-diagnosis (*N* = 975, 467 cases, and 11,320 person-years). The median change time was 11 years (Interquartile range, IQR: 7–15 years) for the pre-diagnosis interval, 5 years (IQR: 4–6 years) for the pre- to post-diagnosis change interval, and 4 years (IQR: 2–4 years) for the post-diagnosis change interval. Figure 2 shows the distribution of changes in alcohol consumption across each change interval. Most women who changed their consumption moved to the next adjacent category. Overall, 74% of participants consumed on average less than one drink/day at baseline. By the end of the pre-diagnosis change interval 11.1% had quit, 17.3% reduced, 19.0% increased, and 52.6% of women were stable in their consumption. In the pre- to post-diagnosis interval most women remained stable in their consumption (53.9%), but 14.4% had quit, 20.2% reduced, and 11.6% increased their consumption. For the post-diagnosis change interval, 56.6% were stable, 9.2% had quit, 17.5% reduced, and 16.6% increased their alcohol consumption.

Participant characteristics by change in alcohol groups for the pre- to post-diagnosis change interval, the interval with the largest sample, are presented in Table 1. The sample was primarily White (98.5%), and college educated (44.3%), with a median age-at-diagnosis of 71 years. There were no notable differences in self-reported race, mammogram history, or family history of breast cancer by change in alcohol group. Compared to stable consumers, women who quit drinking were less educated (33.9% vs. 21.9% less than or equal to high school education), more likely to have at baseline or be diagnosed over the study period with diabetes or CVD comorbidities (24.1% vs. 16.5%), and to report poor or fair perceived health status (12.5% vs. 5.1%). Pre-diagnosis alcohol intake levels varied across change groups with reducers consuming the most (mean: 22.2 g/day) and quitters consuming the least alcohol (mean: 4.6 g/day).

### Change in alcohol consumption and all-cause mortality (ACM)

Associations of alcohol change and ACM among breast cancer survivors are shown in Table 2. Across all change intervals, reduced and increased alcoholic consumption were not associated with ACM. In the pre-diagnosis change interval, there was some evidence of decreased ACM for alcohol cessation compared to stable consumption (HR:0.91, 95%CI: 0.69–1.21), but the confidence intervals were wide and included the null. Pre- to post-diagnosis alcohol cessation was associated with increased risk of ACM compared to stable consumers (HR:1.39, 95%CI: 1.12–1.73). Alcohol cessation post-diagnosis was not associated with ACM (HR:0.98, 95%CI: 0.70–1.35).

Effect modification by baseline comorbidity and self-reported health status were examined (Table 2). In the pre-diagnosis interval, women who quit and did not report poor health indicators showed a nonsignificant suggestion of lower ACM compared to stable consumers (HR:0.87, 95%CI: 0.60–1.26); however, the overlapping confidence intervals and multiplicative interaction p-values indicated no effect modification by health status across all change intervals.

### Sensitivity analyses

The results of restricted subgroup analyses are presented in Table 3. In the pre-diagnosis change interval, no associations were observed in analyses restricted to women without major comorbidities during the corresponding change interval, never smokers, those with good or excellent health, or in those with localized tumors. Among women diagnosed with ER+ tumors, quitting before diagnosis was associated with increased ACM (HR:1.34, 95%CI: 1.01–1.77) compared to stable consumers. Among women consuming less than or equal to one drink/day in the pre-diagnosis interval, increasing consumption was associated with a greater risk of ACM (HR:1.83, 95%CI: 1.02–3.31). The significant association between pre- to post-diagnosis alcohol cessation and ACM persisted in sensitivity analyses. In the subset of women who had sufficient post-diagnosis alcohol measures, we examined sustained pre- to post-diagnosis change (Supplemental Materials, Table 4). The median follow-up duration for assessing sustained post-diagnosis change was 4 years (IQR: 2–4 years). Associations for sustained post-diagnosis reduction or increases were consistent with the initial changes reported; however, sustained cessation was no longer statistically associated with an increase in mortality. Lastly, restricted subgroup analyses for the post-diagnosis change interval resulted in no associations with ACM.

## Discussion

This study is one of the first to prospectively examine the association between change in alcohol consumption, examining reduction and cessation separately, with mortality in a U.S. cohort of breast cancer survivors. Among older postmenopausal breast cancer survivors followed for up to 21 years, reduction or cessation in consumption of alcoholic beverages were not associated with a decrease in ACM risk compared to stable consumers, though associations varied by timing of change. Contrary to our hypothesis, alcohol cessation in the pre- to post-diagnosis period was associated with an increased risk of ACM. This counterintuitive association may be confounded by factors not captured for this change interval, such as overall health status, treatment side-effects, and/or unmeasured comorbidities. In the pre-diagnosis interval, there was a suggestion that alcohol cessation may be associated with decreased ACM compared to stable consumption, although statistical significance was not reached.

We found that among breast cancer survivors who were consuming alcohol before their diagnosis, roughly 86% continued drinking post-diagnosis, with ~ 12% increasing beyond their pre-diagnosis level. Understanding the role of change in alcohol consumption for breast cancer survivors in relation to mortality is of potential clinical significance. Our largely null findings for alcohol reduction and inverse findings for cessation are consistent with what has been observed in other studies [7–9, 11–13, 20]. If alcohol influences mortality through irreversible pathways, behavior modification in later life may not improve survival; consequently, without a clearer understanding of these biological mechanisms and their potential for reversal, it is more difficult to draw conclusions. However, if the impacts of alcohol consumption are reversible then several factors may explain the increased rate of ACM following alcohol cessation in the pre- to post-diagnosis change interval. These include possible insufficient duration of change, later-life timing of change, absence of heavy drinking in the cohort, and lack of information regarding alcohol intake prior to the start of the study. It may also be that alcohol cessation later in life has no or very little impact on survival for women with breast cancer.

In the broader context of health behavior change, studies of smoking cessation and mortality consistently show that earlier cessation (i.e., quitting smoking before age 40) and longer duration of quitting (i.e., 10 or more years of sustained cessation) are important change-related factors associated with improved survival [21–23]. Our study sample consisted of older postmenopausal breast cancer survivors (mean age-at-diagnosis: 71 years) and did not include data on lifetime drinking history. Alcoholic beverage cessation or reduction in a cohort of younger women, could potentially yield stronger associations, as changes could occur earlier and/or be sustained for a longer duration. In a sensitivity analysis, sustained pre- to post-diagnosis cessation showed a weakened association with mortality, supporting the possibility that long-term sustained cessation is needed for a measurable benefit. Additionally, alcohol intake in our sample was low overall, with only approximately 11% of women drinking two or more drinks/day. To assess if a larger change in consumption would impact results, we did a secondary analysis restricted to women who were consuming at least one drink/day. We found that increased pre-diagnosis consumption beyond one drink/day was associated with a greater risk of ACM (HR:1.83, 95%CI: 1.02–3.31).

The observed changes in consumption may be confounded by unknown reasons for changing alcohol consumption. Because these motivations are rarely random, they may independently influence mortality risk. Although we lacked direct data on the reason for change, we were able to stratify analyses by health status based on self-reported perceived health and comorbidity status. We accounted for major comorbidities associated with alcohol and ACM; however, not all comorbidities were included. There were some indications of differences by health status though confidence intervals overlapped, and all included the null. We saw decreased mortality for pre-diagnosis cessation in healthier women, and an elevated risk of mortality for women in poor health (HRs:0.87 and 1.05, respectively). Similarly, in analyses restricted to never smokers, a likely healthier group, the protective associations for cessation were more pronounced than in the entire cohort for both the pre-diagnosis and post-diagnosis intervals. Our findings align with previously published concerns, that those who quit may have risk profiles closer to people who drank previously or those able to continue drinking may be different in several behaviors and overall health than individuals who quit [24–27]. Poor health may modify the association between change in alcohol and ACM, but power was lower for stratified analyses.

The CPS-II cohort’s large sample size, long follow-up, and repeated alcohol measures make it ideal to examine the association between change in alcohol and survival. Our findings must be interpreted considering a few key limitations. Alcohol consumption was self-reported, which may have led to underreporting; however, any resulting misclassification would be non-differential with respect to mortality. Currently, there are no valid objective biomarkers of long-term alcohol consumption. While variations in alcohol assessment across questionnaires may have introduced exposure misclassification, this was likely minimized through the implemented data harmonization procedures. Similarly, although we estimated the proportion of participants who may have quit due to health concerns, we lacked a direct measure of the reason for change. Consequently, some sick quitters may have been misclassified. We did not have data regarding treatment for breast cancer, but we were able to adjust for ER status and stage. Due to a low prevalence of ER- cases, we were unable to stratify by receptor status as planned and instead restricted our final analyses to ER+ cases in a sensitivity analysis [28, 29]. Exploration of post-diagnosis data may be impacted by survival biases, that is, women had to survive several years after diagnosis for there to be multiple measures of post-diagnosis alcohol intake. Our sample had a low prevalence of heavy drinking; we had to collapse higher intake categories to those consuming two or more drinks/day. Lastly, our study population consisted mostly of older White women, which may limit the generalizability of our findings to other groups, including younger women and women of color.

## Conclusion

In this sample of older breast cancer survivors, alcoholic beverage reduction or cessation were not associated with a decrease in all-cause mortality risk. There was suggestion of improved mortality in women who quit before their diagnosis, specifically women who quit in good health. Increased consumption before diagnosis among those already consuming at least one drink per day was associated with poorer survival outcomes. Our results may be limited by uncontrolled confounding, a lack of heavy alcohol consumption in this population, and an insufficient duration of change to observe differences in mortality. Future studies that can harmonize data across multiple cohorts and/or include younger populations may be warranted to examine survival following change in alcohol consumption. Our finding that many women continue to drink after a breast cancer diagnosis highlights the need for continued research on alcohol consumption in this population, particularly given the intersection of challenges for treatment, survivorship, and behavior change.

**Table 1:** CPS-II breast cancers survivors’ baseline (1992/93) characteristics by pre- to post-diagnosis change in alcohol group (*N* = 1,558)

Characteristic	Change in Alcohol Pre- to Post-Diagnosis			
	Stable	Quit	Reduced	Increased
	*N* = 839	*N* = 224	*N* = 314	*N* = 181
	*n (%)*			
**Age at diagnosis** (years), mean ± SD	70.7 ± 7.1	72.1 ± 7.7	71.5 ± 6.8	69.4 ± 7.1
**Education**				
≤ High School Graduate	184 (21.9)	76 (33.9)	73 (23.3)	46 (25.4)
Vocational/Trade School/Some College	267 (31.8)	67 (29.9)	102 (32.5)	53 (29.3)
≥ College Graduate	388 (46.3)	81 (36.2)	139 (44.3)	82 (45.3)
**Race**				
White Participants	827 (98.6)	217 (96.9)	313 (99.7)	177 (97.8)
Black / Other Participants	*	*	*	*
* Missing*	*	*	*	*
**Major Comorbidity Status** ^a^				
None	701 (83.6)	170 (75.9)	254 (80.9)	153 (84.5)
Any, baseline or incident	138 (16.5)	54 (24.1)	60 (19.1)	28 (15.5)
**Mammogram History**				
Ever	811 (96.7)	209 (93.3)	300 (95.5)	174 (96.1)
Never	*	*	*	*
* Missing*	*	*	*	*
**Family History of Breast Cancer**				
Yes	147 (17.5)	36 (16.1)	50 (15.9)	36 (19.9)
No	666 (79.4)	179 (79.9)	250 (79.6)	137 (75.7)
* Missing*	26 (3.1)	9 (4.0)	14 (4.5)	8 (4.4)
**Postmenopausal Hormone Use**				
Ever	487 (58.1)	111 (49.6)	193 (61.5)	104 (57.5)
Never	340 (40.5)	111 (49.6)	119 (37.9)	74 (40.9)
* Missing*	12 (1.4)	2 (0.9)	2 (0.6)	3 (1.7)
**Physical Activity (hrs/wk)**, median (25th-75th)^b^	8.0 (3.5–18.0)	7.5 (3.5–15.0)	10.5 (3.5–18.5)	8.0 (3.5–21.0)
**Body Mass Index** (kg/m^2^), mean ± SD	25.0 ± 4.2	26.0 ± 4.5	24.6 ± 3.5	24.4 ± 4.5
**Pre-diagnosis Alcohol Intake** (g/day), mean ± SD	9.8 ± 11.9	4.6 ± 7.7	22.2 ± 12.9	9.8 ± 6.4
**Smoking Status**				
Never	352 (42.0)	125 (55.8)	112 (35.7)	58 (32.0)
Current	77 (9.2)	21 (9.4)	27 (8.6)	27 (14.9)
Former	410 (48.9)	78 (34.8)	175 (55.7)	96 (53.0)
**Tumor Stage**				
Localized	651 (77.6)	167 (74.6)	244 (77.7)	143 (79.0)
Regional	188 (22.4)	57 (25.5)	70 (22.3)	38 (21.0)
**ER Status**				
Positive (+)	733 (87.4)	200 (89.3)	274 (87.3)	155 (85.6)
Negative (-)	106 (12.6)	24 (10.7)	40 (12.7)	26 (14.4)
**Perceived Health Status** ^c^				
Poor-Fair	43 (5.1)	28 (12.5)	25 (8.0)	7 (3.9)
Good-Excellent	534 (63.7)	127 (56.7)	209 (66.6)	97 (53.6)
* Missing*	262 (31.2)	69 (30.8)	80 (25.5)	77 (42.5)

CPS-II= Cancer Prevention Study-II; SD= standard deviation; ER= estrogen receptor status; *= sparse data

^a^Comorbidity index includes diabetes and CVD status diagnosed at baseline or incident during the time period

^b^Missing data for continuous variables: physical activity *n* = 7

^c^Perceived health status at post-diagnosis survey; variable not included in all surveys

**Table 2:** Change in alcohol consumption pre-diagnosis, pre- to post-diagnosis, and post-diagnosis and all-cause mortality among women consuming alcohol at baseline, subsequently diagnosed with breast cancer, CPS-II (1992–2020)

Change Interval	Events	Person-years	All-Cause Mortality	
			Model 1^a^	Model 2^b^
			HR (95% CI)*	
**Pre-Diagnosis Change**, *N* = 1,330	608	17,059		
Per 7 g/day decrease^c^	179		0.92 (0.85–0.99)	0.98 (0.81–1.20)
Stable Intake	324	8,990	1.00	1.00
Reduced^d^	102	2,945	0.94 (0.75–1.17)	1.07 (0.82–1.38)
Increased	105	3,468	0.89 (0.71–1.10)	1.06 (0.84–1.33)
All Quit	77	1,656	1.38 (1.08–1.77)	0.91 (0.69–1.21)
Quit, Yes Poor Health^e^	34		2.02 (1.42–2.89)	1.05 (0.72–1.54)
Quit, No Poor Health	43		1.11 (0.81–1.52)	0.87 (0.60–1.26)
Quit x Poor Health, p-value				0.47
**Change Pre- to Post-Diagnosis**, *N* = 1,558	750	19,173		
Per 7 g/day decrease^c^	279		1.00 (0.94–1.06)	0.99 (0.92–1.06)
Stable Intake	383	10,573	1.00	1.00
Reduced^d^	154	3,666	1.08 (0.89–1.30)	1.03 (0.82–1.28)
Increased	88	2,482	0.97 (0.77–1.23)	1.08 (0.85–1.39)
All Quit	125	2,451	1.37 (1.12–1.68)	1.39 (1.12–1.73)
Quit, Yes Poor Health^e^	47		1.85 (1.36–2.52)	1.68 (1.22–2.33)
Quit, No Poor Health	78		1.19 (0.93–1.52)	1.34 (1.04–1.72)
Quit x Poor Health, p-value				0.56
**Post-Diagnosis Change**, *N* = 975	467	11,320		
Per 7 g/day decrease^c^	138		1.16 (1.02–1.31)	0.99 (0.74–1.33)
Stable Intake	254	6,476	1.00	1.00
Reduced^d^	82	1,994	1.02 (0.80–1.31)	1.11 (0.83–1.47)
Increased	75	2.025	0.94 (0.72–1.21)	0.99 (0.74–1.31)
All Quit	56	825	1.41 (1.05–1.90)	0.98 (0.70–1.35)
Quit, Yes Poor Health^e^	24		1.45 (0.95–2.22)	0.89 (0.55–1.42)
Quit, No Poor Health	32		1.38 (0.95-2.00)	1.03 (0.70–1.53)
Quit x Poor Health, p-value				0.59

*Hazard ratios (HR) and 95% confidence intervals (CI)

^a^Age at diagnosis adjusted model

^b^Model additionally adjusted for prior alcohol intake, time difference between surveys, tumor stage, estrogen receptor status, smoking (pack years*smoking status), comorbidities incident or at baseline (diabetes and/or CVD)

^c^Only participants who quit or reduced alcohol consumption

^d^Not including those who quit

^e^Poor health defined by perceived health status and comorbidity status (diabetes and/or CVD) at baseline or incident up until the end of the change interval; comorbidities not adjusted for in model

**Table 3:** Change in alcohol consumption and all-cause mortality restricted to never smokers, those without major comorbidities, those who perceived their health status as good-excellent, ER + tumors, or localized breast tumors

Change Interval	Event/Total	Change in Alcohol Consumption Group			
		Stable	Quit	Reduced	Increased
		All-Cause Mortality, HR (95%CI)^a^			
**Among Never Smokers at Baseline**					
Pre-Diagnosis Change	237/569	1.00	0.71 (0.46–1.09)	0.87 (0.53–1.41)	0.96 (0.64–1.44)
Change Pre- to Post-Diagnosis	291/647	1.00	1.50 (1.11–2.04)	1.16 (0.77–1.75)	1.13 (0.73–1.76)
Post-Diagnosis Change	183/406	1.00	0.94 (0.56–1.60)	1.22 (0.65–2.28)	1.05 (0.67–1.64)
**Among Those Without Poor Health Indicators** ^b^					
Pre-Diagnosis Change	466/1107	1.00	0.90 (0.61–1.32)	1.01 (0.75–1.35)	1.00 (0.77–1.29)
Change Pre- to Post-Diagnosis	536/1217	1.00	1.53 (1.17–2.00)	1.16 (0.89–1.52)	1.33 (1.01–1.74)
Post-Diagnosis Change	289/671	1.00	0.82 (0.51–1.32)	1.07 (0.75–1.53)	1.08 (0.77–1.53)
**Among Those Consuming ≥ 1 drink/day at Time 1**					
Pre-Diagnosis Change	160/345	1.00	1.71 (0.57–5.12)	1.36 (0.93–1.99)	1.83 (1.02–3.31)
Change Pre- to Post-Diagnosis	209/445	1.00	1.37 (0.60–3.13)	0.98 (0.70–1.37)	1.37 (0.74–2.53)
Post-Diagnosis Change	108/240	1.00	1.24 (0.27–5.76)	1.54 (0.92–2.56)	2.01 (0.84–4.81)
**Estrogen Receptor Positive (ER +) Tumors**					
Pre-Diagnosis Change	534/1176	1.00	1.34 (1.01–1.77)	1.19 (0.91–1.56)	1.00 (0.78–1.29)
Change Pre- to Post-Diagnosis	652/1362	1.00	1.54 (1.22–1.93)	1.14 (0.91–1.44)	1.23 (0.96–1.60)
Post-Diagnosis Change	406/851	1.00	0.90 (0.63–1.29)	1.23 (0.90–1.68)	1.02 (0.75–1.38)
**Localized Stage Tumors**					
Pre-Diagnosis Change	458/1039	1.00	1.25 (0.91–1.72)	1.09 (0.81–1.47)	1.17 (0.90–1.52)
Change Pre- to Post-Diagnosis	559/1205	1.00	1.49 (1.17–1.90)	1.06 (0.83–1.36)	1.10 (0.84–1.44)
Post-Diagnosis Change	355/752	1.00	0.98 (0.68–1.41)	0.96 (0.69–1.33)	0.80 (0.58–1.10)

*Hazard ratios (HR) and 95% confidence intervals (CI)

^a^Models adjusted for prior alcohol intake, time difference between surveys, age at diagnosis, stage, estrogen receptor status, smoking (pack years*smoking status), comorbidities (diabetes and/or CVD). The covariate of intertest was not adjusted for in that model

^b^Poor health defined by perceived health status and comorbidity status (diabetes and/or CVD) at baseline or incident up until the end of the change interval

## Supplementary Information

Below is the link to the electronic supplementary material.

Supplementary Material 1

## Data Availability

Data are available from the American Cancer Society (ACS) by following the ACS Data Access Procedures (https://www.cancer.org/content/dam/cancer-org/research/epidemiology/cancer-prevention-study-data-access-policies.pdf) for researchers who meet the criteria for access to confidential data. Please email cohort.data@cancer.org to inquire about access.
