# Supplementary material for: Change in alcohol consumption and all-cause mortality among postmenopausal women diagnosed with breast cancer
Source: Breast Cancer Res Treat. 2026 Oct 23;220(1):14. doi: 10.1007/s10549-026-08113-1 (PMC13694358; doi:10.1007/s10549-026-08113-1)
Supplement: Supplementary file 1 [file 10549_2026_8113_MOESM1_ESM.docx]

**Supplemental Figure 1.** Study Sample Flowchart.

*Covariates Included: stage, education, age-at-diagnosis, pack-years smoked, comorbidity data, body mass index, and estrogen receptor status.

CPS-II postmenopausal women with verified invasive breast cancer with complete 1992 baseline data

**N=5,214**

Excluded women who were not current drinkers, did not have at least two measures of alcohol intake, and were diagnosed with late stage breast cancer

N= 2,665

N=24 late stage

**Post-diagnosis Interval**

Exclude those without two post-diagnosis measures

N= 1,119

Exclude if died within 1 year of change

N= 20

Exclude missing covariate data*

N= 411

**Total Analytic Sample, N=975**

**Pre- to Post-diagnosis Interval**

Exclude those without a pre- and post-diagnosis measures

N= 465

Exclude if died within 1 year of change

N= 20

Exclude missing covariate data*

N= 410

**Total Analytic Sample, N=1,558**

**Pre-diagnosis Interval**

Exclude those without baseline and pre-diagnosis measures

N= 988

Exclude if died within 1 year of dx

N= 2

Exclude missing covariate data*

N= 205

**Total Analytic Sample, N=1,330**

Total Sample

**N=2,525**

| **Supplemental Table 1.** Alcohol exposure assessment by questionnaire. | |
| --- | --- |
| **Survey name**, year | **Alcohol Related Questions** |
| **CPS-II Mortality**  1982 |  |
| **CPS-II Nutrition Study**  1992,  *baseline* |  |
| **CPS-II Nutrition Study Follow-up**  *2003* |    |
| **CPS-II Nutrition Study** **Follow-up**  *2007* |  |
| **CPS-II Nutrition Study** **Follow-up**  *2013* |  |

| **Supplemental Table 2.** Total sample size and frequency of participants by change period and change group. | | | |
| --- | --- | --- | --- |
|  | **Pre-Diagnosis Change** | **Change Pre- to Post-Diagnosis** | **Post-Diagnosis Change** |
| Stable | 700 | 839 | 552 |
| All Quit | 148 | 224 | 90 |
| Quit, No Poor Health | 100 | 155 | 53 |
| Quit, Yes Poor Health | 48 | 69 | 37 |
| Reduced | 230 | 314 | 171 |
| Increased | 252 | 181 | 162 |
| Total | 1,330 | 1,558 | 975 |
|  | | | |

| **Supplemental Table 3.** Associations between change pre- and post-diagnosis, pre-diagnosis change, and post-diagnosis alcohol change and breast cancer-specific mortality among women diagnosed with breast cancer in the CPS-II (1992-2020). | | | |
| --- | --- | --- | --- |
| Change Interval | Events | **Breast Cancer-Specific Mortality** | |
|  |  | Model 1^a^ | Model 2^b^ |
|  |  | HR (95% CI) | |
| **Pre-Diagnosis Change,** N=1,330 | 100 |  |  |
| Per 7 g/day decrease^c^ | 26 | 0.74 (0.54-1.03) | 0.80 (0.33-1.93) |
| Stable Intake | 63 | 1.00 | 1.00 |
| Reduced^d^ | 10 | 0.48 (0.24-0.92) | 0.59 (0.27-1.31) |
| Increased | 11 | 0.46 (0.25-0.87) | 0.52 (0.26-1.03) |
| All Quit | 16 | 1.27 (0.73-2.20) | 0.63 (0.29-1.38) |
| **Change Pre- and Post-Diagnosis,** N=1,558 | 130 |  |  |
| Per 7 g/day decrease^c^ | 47 | 0.88 (0.73-1.06) | 0.78 (0.58-1.06) |
| Stable Intake | 69 | 1.00 | 1.00 |
| Reduced^d^ | 26 | 1.02 (0.65-1.60) | 1.17 (0.65-2.10) |
| Increased | 14 | 0.87 (0.49-1.56) | 1.06 (0.58-1.93) |
| All Quit | 21 | 1.17 (0.71-1.91) | 0.98 (0.59-1.62) |
| **Post-Diagnosis Change,** N=975 | 71 |  |  |
| Per 7 g/day decrease^c^ | 21 | 0.77 (0.49-1.21) | 1.04 (0.40-2.67) |
| Stable Intake | 39 | 1.00 | 1.00 |
| Reduced^d^ | 9 | 0.72 (0.35-1.48) | 0.83 (0.37-1.87) |
| Increased | 11 | 0.91 (0.46-1.78) | 0.71 (0.32-1.57) |
| All Quit | 12 | 2.10 (1.09-4.06) | 1.49 (0.65-3.39) |
| Note: HR= hazard ratio; CI= 95% confidence interval | | | |
| ^a^Age adjusted model. | | | |
| ^b^Model adjusted for prior alcohol intake, time difference between surveys, age at diagnosis, stage, estrogen receptor status, smoking (pack years*smoking status), comorbidities incident or at baseline(diabetes and/or CVD). | | | |
| ^c^Only included participants who quit or reduced alcohol consumption. | | | |
| ^d^Not including those who had a 100% reduction in use, quit. | | | |

| **Supplemental Table 4.** Sensitivity Analysis: Associations between sustained post-diagnosis change and all-cause mortality among women diagnosed with breast cancer in the CPS-II. | | | |
| --- | --- | --- | --- |
| Change Interval | Events | **All-Cause Mortality** | |
|  |  | Model 1^a^ | Model 2^b^ |
|  |  | HR (95% CI) | |
| **Sustained Post-Diagnosis Change,** N=941^c^ | 460 |  |  |
| Sustained Stable Intake | 153 | 1.00 | 1.00 |
| Sustained Any Reduction (R +Q) | 79 | 1.27 (0.96-1.67) | 1.12 (0.83-1.50) |
| Sustained Reduction | 40 | 1.06 (0.74-1.50) | 1.07 (0.73-1.57) |
| Sustained Quit | 39 | 1.58 (1.11-2.25) | 1.17 (0.79-1.75) |
| Sustained Increase | 17 | 0.70 (0.43-1.16) | 1.08 (0.64-1.80) |
| Newly Reduced | 83 | 1.06 (0.81-1.39) | 1.18 (0.88-1.59) |
| Newly Quit | 55 | 1.61 (1.17-2.21) | 1.18 (0.84-1.64) |
| Newly Increased | 73 | 0.97 (0.73-1.28) | 1.25 (0.92-1.70) |
| Note: HR= hazard ratio; CI= 95% confidence interval | | | |
| ^a^Age adjusted model. | | | |
| ^b^Model adjusted for prior alcohol intake, time difference between surveys, age at diagnosis, stage, estrogen receptor status, smoking (pack years*smoking status), comorbidities incident or at baseline (diabetes and/or CVD).  ^C^Median time of sustained post-diagnosis change: 4 years (IQR: 2-4 years). | | | |

| **Supplemental Table 5.** Sensitivity Analysis: Associations between change in alcohol consumption and all-cause mortality stratified by median follow-up time breast cancer survivors in the CPS-II. | | | | | | | |
| --- | --- | --- | --- | --- | --- | --- | --- |
| Change Interval | Events /  Total | | **Change in Alcohol Consumption Group** | | | | |
|  |  |  | Stable | Quit | Reduced | Increased | Per 7g/day decrease^a^ |
|  |  |  | All-Cause Mortality, HR (95%CI)^b^ | | | | |
| Follow-up time < 12.6 years^c^ | | | | | | | |
| Pre-Diagnosis Change | 396/672 | | 1.00 | 0.94 (0.68-1.31) | 1.10 (0.80-1.51) | 0.94 (0.69-1.30) | 0.99 (0.77-1.28) |
| Change Pre- and Post-Diagnosis | 499/775 | | 1.00 | 1.28 (0.99-1.64) | 0.97 (0.73-1.27) | 0.99 (0.73-1.36) | 1.00 (0.92-1.10) |
| Post-Diagnosis Change | 358/526 | | 1.00 | 0.97 (0.68-1.40) | 0.97 (0.69-1.38) | 0.97 (0.70-1.35) | 0.99 (0.74-1.33) |
| Follow-up time ≥ 12.6 years^c^ | | | | | | | |
| Pre-Diagnosis Change | 212/658 | 1.00 | | 1.27 (0.75-2.18) | 0.95 (0.58-1.56) | 0.93 (0.65-1.32) | 0.91 (0.59-1.40) |
| Change Pre- and Post-Diagnosis | 251/783 | 1.00 | | 1.15 (0.73-1.80) | 1.01 (0.69-1.47) | 0.92 (0.61-1.40) | 0.91 (0.81-1.03) |
| Post-Diagnosis Change | 109/449 | 1.00 | | 1.61 (0.69-3.74) | 0.83 (0.43-1.60) | 1.21 (0.67-2.18) | — |
| Note: HR= hazard ratio; CI= 95% confidence interval | | | | | | | |
| ^a^Only included participants who quit or reduced alcohol consumption; sample sizes vary. | | | | | | | |
| ^b^Model adjusted for prior alcohol intake, time difference between surveys, age at diagnosis, stage, estrogen receptor status, smoking (pack years*smoking status), comorbidities incident or at baseline(diabetes and/or CVD). | | | | | | | |
| ^c^Stratified by the median follow-up time for the pre- to post-diagnosis change interval. | | | | | | | |
